# Targeting miR-9 in Glioma Stem Cell-Derived Extracellular Vesicles: A Novel Diagnostic and Therapeutic Biomarker

**DOI:** 10.1016/j.tranon.2022.101451

**Published:** 2022-05-19

**Authors:** Liangyuan Geng, Jinjin Xu, Yihao Zhu, Xinhua Hu, Yong Liu, Kun Yang, Hong Xiao, Yuanjie Zou, Hongyi Liu, Jing Ji, Ning Liu

**Affiliations:** a Department of Neurosurgery, The First Affiliated Hospital of Nanjing Medical University, Nanjing 210009, People's Republic of China; b Clinical Molecular Diagnostic Laboratory, The Second Affiliated Hospital of Nanjing Medical University, Nanjing 210003, People's Republic of China; c Department of Neurosurgery, Affiliated Nanjing Brain Hospital, Nanjing Medical University, Nanjing 210029, People's Republic of China; d Department of Neuro-Psychiatric Institute, Affiliated Nanjing Brain Hospital, Nanjing Medical University, Nanjing 210029, People's Republic of China

**Keywords:** Glioblastoma, Cerebrospinal fluid, Extracellular vesicles, miR-9, Glioma stem cell, DACT3, GBM, glioblastoma multiforme, GSC, glioma stem cell, CSF, cerebrospinal fluid, CNS, central nervous system, BBB, blood-brain barrier, EVs, extracellular vesicles, miRNA, microRNA, RT-qPCR, realtime quantitative polymerase chain reaction, RNA-Seq, RNA sequencing, PBS, phosphate-buffered saline, NTA, nanoparticle tracking analysis, TEM, Transmission electron microscopy, GO, Gene Oncology, KEGG, Kyoto Encyclopedia of Genes and Genomes, RIPA, radio-immunoprecipitation assay, ROC, Receiver Operating Characteristics, AUC, area under curve, DACT3, Dapper antagonist of beta-catenin 3

## Abstract

**Background:**

Glioblastoma (GBM) is a lethal brain tumor with no effective strategies in early diagnosis and treatment. This study was aimed to assess the miRNA expression profiles in EVs from CSF and tissue of glioblastoma patients to identify significantly upregulated miRNAs and investigate the underlying neoplastic mechanisms.

**Methods:**

EVs were measured by TEM and NTA assays. Differentially regulated miRNAs were measured using RNA sequencing in GBM CSF EVs and in GBM tissues compared with controls. RT-qPCR was employed to analyze miRNA and gene expression. Luciferase report assay was used to investigate gene target of miR-9. The proliferation ability was detected by EdU and CCK-8 experiment while cell migration was measured by transwell and wound healing assay.

**Results:**

The expression level of miR-9 was significantly higher in GBM CSF EVs and tissues than controls (p = 0.038). The area under curve for CSF EV miR-9 was 0.800 (95% CI: 0.583–1.000, p = 0.033). The expression of miR-9 was significantly higher in Glioma stem cells (GSCs) and GSC-derived EVs than in glioblastoma cells. GSC-derives EVs could promote GBM growth and migration Moreover, inhibition of miR-9 in GSCs showed the reverse anti-tumor effects through secreted EVs. MiR-9 could bind to the 3’UTR region of DACT3 and suppress its expression. The miR-9/DACT3 axis might attribute to GBM malignant phenotype.

**Conclusion:**

MiR-9 in CSF EVs may act as a novel diagnostic biomarker for GBM and targeting miR-9 by GSC-derived EVs may be a specific and efficient strategy for GBM biotherapy.

## Highlights

• MiR-9 was upregulated in CSF EVs of glioblastoma patients.
• The expression of miR-9 was increased in GSCs and GSC-derived EVs.
• Inhibition of miR-9 in GSC-EVs suppressed the GBM malignant phenotypes via the regulation of DACT3.

## Introduction

Glioblastoma multiforme (GBM) is the most common and lethal primary brain tumor in adults [1]. Even with aggressive surgery and combined radiotherapy and chemotherapy [2], the median survival of GBM patients is approximately 14 months [3]. Owing to its high proliferation and invasion ability, GBM always grow to a large size before diagnosis [4]. Moreover, current monitoring strategies fail to give insights into molecular evolution in response to therapeutic interventions [5]. Hence, novel biomarkers are urgently needed for early diagnosis of GBM and monitoring of treatment response.

Cerebrospinal fluid (CSF) is considered an appropriate source of biomarkers for central nervous system (CNS) diseases because of its direct contact with the brain, which often reflects CNS pathophysiological changes [6], [7], [8], [9]. Extracellular vesicles (EVs) are membrane-contained vesicles enclosing proteins and distinct species of nucleic acids released from tumor cells into the microenvironment. EVs and the contents they carry play roles in maintaining cellular homeostasis and intercellular communication [10]. Moreover, EVs are present in CSF, and their lipid bilayer protects intrinsic cargo from an otherwise hostile biofluid environment replete with RNases [11]. MicroRNAs (miRNAs) are small non-coding RNAs known to be involved in GBM initiation and progression [9]. MiRNAs are enriched in CSF EVs and can be detected by real-time quantitative polymerase chain reaction (RT-qPCR) [12,13]. There is a growing interest in using EV-derived miRNAs released from GBM cells into CSF as a diagnostic biomarker.

Previous studies found that several miRNAs in CSF EVs of GBM patients were upregulated compared to controls [13,14]. However, there is still no appropriate diagnostic marker for GBM. Here, we utilize RNA-sequencing (RNA-Seq) and bioinformatics methods to assay EV miRNAs’ repertoire in the CSF of patients with GBM compared to healthy controls. Furthermore, we describe a novel EV miR-9 in CSF that is highly predictive of preoperative GBM diagnosis.

Cancer stem cells have been emerging as a promising target for cancer research and treatment owing to its dominant responsibility for tumor initiation, differentiation, resistance to existing therapies and recurrence [15]. Glioma stem cell (GSC) is a subtype of GBM located in SVZ, which are characterized by the specific surface markers [16], such as CD133, CD15, SOX2 and Nestin. Current opinions about GSC mainly focus on its favorable function in communication with tumor microenvironment, which contributes to GBM treatment resistance and inevitable recurrence [17]. However, the interaction mechanism between GSCs and their progeny or glioblastoma cells remains unknow.

Ample data indicate that GSCs regulate peripheral environment via secretion of EVs and soluble molecules [18], as well as by direct cell contacts. GSC-derived EVs could be detected in CSF by virtue of the disruption of the BBB in GBM patients. Collectively, GSCs served as a reliable resource of secreted exosomes, and glioma cells are able to receive EVs [19]. This may lead to the hypothesis that GSCs exert influence on glioma cells through GSC-derived EVs. As documented, the crucial neoplastic effects of tumor secreted exosomes can be attributed to their miRNA cargo, which is protected by the membrane-contained vesicles [20]. Hence, in this study, we detected the content of miR-9, which was significantly increased in GBM patients’ CSF, GSCs and GSC-derived EVs. Furthermore, we treated the glioblastoma cell lines with GSC supernatant to analyze the role of EV-contained miR-9 and the underlying molecular pathway in GBM proliferation and migration.

## Materials and methods

### Patients and samples

A drainage tube was inserted into the subarachnoid space of GBM patients using the lumbar puncture method before anesthesia. The drainage tube remained closed until cranial surgery. CSF was collected from the cistern of control patients during some meningioma surgeries, which employed CSF drainage for better tumor exposure. We collected 30–50 mL of CSF at 4°C for each patient. Tumor and brain tissues accessed from the Tumor and Tissue Bank at Nanjing Brain Hospital.

**EVs isolation.** EVs were isolated from GBM, CSF, and GSC culture medium using differential centrifugation at 4°C. Briefly, the CSF and GSC supernatant were initially centrifuged at 3,500 g for 10 min to remove suspended cells. The supernatant was then centrifuged two times at 4,500 g for 10 min each, followed by 10,000 g for 30 min to remove cellular debris. The pellet was re-suspended in phosphate-buffered saline (PBS) and further centrifuged at 100,000 g for 60 min. The 100,000 g pellet was centrifuged again after dilution with PBS. The final CSF and GSC-derived EV pellets were used immediately or re-suspended in PBS and stored at -80°C.

### Transmission electron microscopy (TEM)

CSF and GSC-derived EVs (10 μg) were re-suspended in PBS and loaded onto Formvar/Carbon-coated grids (Ted Pella Inc., Redding, CA, USA) fixed in 2.5% glutaraldehyde, stained with 2% uranyl acetate, and visualized with LEO 912AB Omega electron microscope (FEI Tecnai G2 Spirit Bio TWIN, USA).

### Western blot analysis

Isolated EVs were digested with RIPA lysis buffer and centrifuged for the supernatant protein. The supernatants were mixed with Loading Buffer and heated. The samples loaded onto 10% SDS-PAGE gels. Proteins were transferred onto PVDF membranes, blocked with 5% non-fat milk for 2 h, and incubated overnight with primary antibody against CD63 (Abcam, ab59479), GAPDH (Abcam, ab181602), Hsp70 (Abcam) at 4°C. The membranes were washed three times with PBST and incubated with HRP-conjugated secondary antibody (Bioss, bs-40295G-HRP) for 2 h. The detection was imaged using a chemiluminescence instrument (Tanon 4200, Shanghai, China).

### Nanoparticle tracking analysis (NTA)

Size determination of CSF and GSC-derived EVs was analyzed using ZetaView (Particle Matrix GmbH, Microtrac, Meerbusch, Germany).

### RNA sequencing (RNA-Seq)

Using TRIzol, total RNA was extracted from CSF EVs and glioma tissues using the miRNeasy Micro Kit (Qiagen, Hilden, Germany). Small (i.e.,18–30 nt) RNAs were enriched by polyacrylamide gel electrophoresis (PAGE). Then the 3′ adapters added, and the 5′ adapters were ligated. The products were reverse transcribed using PCR amplification. Their 140–160 bp size products were enriched to generate a cDNA library and finally sequenced on an Illumina HiSeqTM2500 system (San Diego, CA, USA).

### Bioinformatics analysis

To obtain high-quality reads, the raw data was further filtered according to the following rules: reads containing more than one low quality (Q-value ≤ 20) base or unknown nucleotides (N); reads without 3′ adapters or having 5′ adapters; reads containing 3′ and 5′ adapters but no small RNA fragment between them; reads containing poly A in small RNA fragment; reads shorter than 18 nt. Then the tiny RNAs were aligned and identified. Significantly differentially expressed miRNAs were identified using edgeR software default parameters with a fold change ≥2 and P-value < 0.05 in comparison. The potential target genes of the dysregulated miRNAs were then undergoing GO and KEGG analysis. We map the candidate genes to each term in the GO and KEGG database and calculate the number of genes per term, so as to obtain the list of genes with a certain GO function and KEGG pathway. Hypergeometric tests are then applied to identify GO and KEGG entries that are significantly enriched in candidate genes compared to the entire genome background.

### Real-time quantitative polymerase chain reaction (RT-qPCR)

Total RNA was isolated from glioblastoma cells, exosomes and cells using Trizol reagent (Thermo Scientific, USA). The relative RNA concentration and quality were measured on a DU800 UV spectrophotometer (Beckman Counter, USA). cDNA was synthesized using a cDNA synthesis kit (Yeasen, Shanghai, China). qPCR was performed using qPCR SYBR Green Master Mix on a Lightcycler (QuantStudio 5, ABI, USA). The data were calculated using the comparative threshold cycle relative-quantification method.

### Animals

All experimental animal procedures were reviewed and approved by the Ethics Committee for Animal Experimentation at Nanjing Medical University (Nanjing, China) and performed in accordance with the Guide for the Care and Use of Laboratory Animals by the National Institutes of Health. Male mature BALB/c nude mice were purchased from the Animal Center of Nanjing Medical University. The mice were housed at the animal feeding institution, where room temperature, humidity were monitored, and 12 h light/dark cycle was followed.

### Isolation and characterization of glioma stem cells (GSCs)

The detailed experimental protocol for GSC isolation is described elsewhere [21]. Briefly, GSCs were isolated and purified from GBM patient tissues and the extracted GSC primary spheroids. The GSCs were incubated in the DMEM/F12 (Gibco, Waltham, MA, USA) medium supplemented with B27(1:50, Invitrogen, Carlsbad, CA, USA), N2(1:100, Invitrogen), 10 ng/mL epidermal growth factor and 10 ng/mL fibroblast growth factor (FGF, Invitrogen).

The GSCs morphology was photographed and recorded in detail. The GSCs were labeled with different monoclonal antibodies (SOX2 and Nestin) for immunofluorescence analysis.

### Glioblastoma cell culture

Two human glioblastoma cell lines (U87 and U251) were purchased from the Cell Bank of Chinese Academy of Sciences (Shanghai, China). The cells were cultured in DMEM medium supplemented with 10% FBS and 1% penicillin/streptomycin (Thermo Fisher Scientific, USA). The incubator was maintained at 37°C in a concentration of 95% humidity and 5% CO_2_.

### Cell co-culture system

A total of 1 × 10^6^ U87 or U251 cells were seeded on the lower chamber of a six-well co-culture plate, while an equal amount of 10 μg GSC-derived EVs were added to the upper chamber. The six-well plate was cultured in a 37°C incubator for 24 h. Finally, glioblastoma cell lines were harvested for further analysis.

### MiRNA transfection

The amount of 5 × 10^5^ GSCs were seeded on the six-well plate for 24 h. Then, 5 μL Lipofectamine 6000 (Beyotime, Shanghai, China) was blended with 50 μL serum-free Opti-MEM medium for 5 min. Simultaneously, a 100 pmol miR-9 mimic or inhibitor were added into the 50 μL Opti-MEM medium. Then this medium was mixed and allowed to rest for 20 min. Lastly, the 100 μL mixture was taken to the GSCs culture medium and incubated for 24 h. The sequence of Has-miR-9-5p mimic: UCUUUGGUUAUCUAGCUGUAUGA—AUACAGCUAGAUAACCAAAGAUU; and Has-miR-9-5p inhibitor: UCAUACAGCUAGAUAACCAAAGA.

### Intracranial xenograft model

Four-week-old male BALB/c nude mice were randomly divided into 2 groups (sh-NC and sh-miR-9). GSCs were transfected with stably expressed sh-NC and sh-miR-9 luciferase lentivirus. Intracranial tumor growth was measured by in vivo fluorescence imaging. Each mouse was anesthetized by initial 2% isoflurane in 100% O_2_ and maintained in 1% isoflurane. The mice were then given an intraperitoneal injection of D-luciferin (50 mg/mL). Xenografts were imaged by means of Caliper Lumina system (Caliper Life Science, Waltham, MA, USA). Mouse survival time was monitored until death.

### EdU (5-ethynyl-2′-deoxyuridine) assay

The cell proliferation analysis was conducted by EdU assay. U87 and U251 cells were co-cultured with different groups of GSC-derived exosomes in a concentration of 10 μg/mL. After incubation for 24 h, 37°C, 20 μM EdU reagent was added and reacted for 2 h. Then the culture medium was discarded and the sample was washed with PBST. Cells were fixed with paraformaldehyde for 15 min. We added click addition solution and incubated for 30 min. Then 1× Hoechst 33342 was administered for 10 min in the dark. We randomly selected six fields of view by fluorescence microscope (Carl Zeiss Meditec AG, Jena, Germany) with two observers blind to group assignments.

### Cell viability assay

Cell Counting Kit-8 (CCK-8; Dojindo, Kumamoto, Japan) was employed to analyze cell proliferation. Equal amount of about 1 × 10^3^ cells was seeded in the 96-well plates and cultivated for several days. 10% CCK-8 dilutions were added to cell medium in every time point and reacted for 2 h. Subsequently, the plates were analyzed with Thermo Fisher microplate reader at the value of 450 nm wavelength (OD450). All the tests were performed at least 3 times.

### Migration assay

Transwell assays were employed to evaluate the migration captivity. Briefly, 2×10^5^ cells in 200 μL DMEM, supplemented with or without EVs, were seeded onto the upper chamber of 6.5 mm transwells (Corning Incorporated, Corning, NY, US). After incubation for 20 h, the interior U87 or U251 cells of the chamber, which did not penetrate the membrane, were removed. After that, the chambers were fixed with paraformaldehyde and then stained with crystal violet. Finally, the migrating cells were measured in five microscopic fields (200×) (Carl Zeiss, Germany).

### Wound healing assay

Equal number of glioblastoma cells were seeded onto the 6-well plates and incubated. The wound was made by tip scratching after cells reaching a density about 90% confluence and then cultured for 24 h to measure wound closure.

### Dual luciferase reporter assay

The predicted binding sequence of DACT3 3′UTR region and the mutate sequence were amplified and cloned into luciferase reporter plasmid (PGL3-DACT3 WT and PGL3-DACT3 mut). The luciferase reporter plasmids were co-transfected with miR-9 mimics. The PGL3-empty vector was used as negative controls. After reaction for 48 h, the luciferase density was examined by the Dual Luciferase Reporter Assay Kit (Vazyme, Nanjing, China) according to the manufacturers’ protocol.

### Statistical analysis

Statistical analyses were performed using GraphPad Prism 5 (GraphPad Software, CA, US) and R software version 3.2.1. Student's t-test was used to determine significant differences between EV miRNAs derived from normal and glioma CSF. A two-sided P-value < 0.05 was considered statistically significant.

## Results

### Patient characteristics

We collected CSF specimens from 10 patients diagnosed with GBM and from 8 controls without brain tumors. Detailed information on GBM and control patients is shown Table 1.

**Table 1:** Characteristics of GBM and control patients.

	**Characteristic**		
**GBM patients**			
	Cases (n)		10
	Age (y)		55.8 ± 15.4
	Gender (female/male)		6/4
	Disease duration (d)		18.70 ± 9.50
	Tumor size(mm)		40.19 ± 12.28
	IDH1 mutation		2
	IDH2 mutation		0
	1p/19q mutation		3
	TERT gene promoter mutation		6
	MGMT methylated		3
	BRAF 15 mutation		2
**Control patients**			
	Cases (n)		8
	Age (y)		61.55 ± 20.53
	Gender (female/male)		5/3

GBM, glioblastoma

### Characterization of CSF EVs

**Fig. 1:**
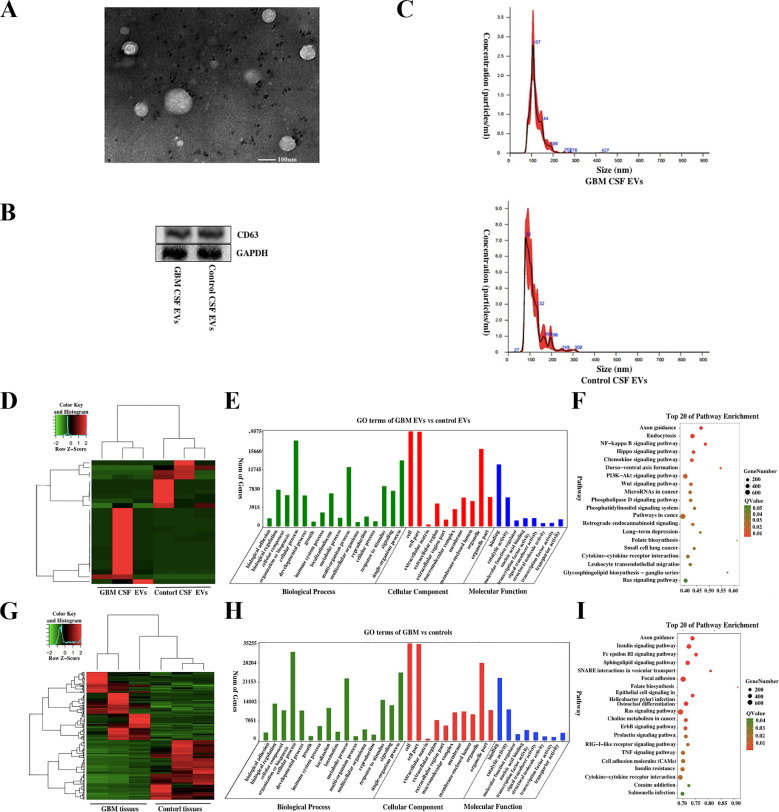
Characterization of EVs isolated from CSF. (A) Transmission electron microscopy of CSF EVs; scale bars: 100 nm. (B) The exosomal marker CD63 was verified using western blot analysis. (C) The size and concentration of GBM and control CSF EVs were assessed using nanoparticle tracking analysis. Characteristics of differential miRNA profiles in CSF and tissue EVs. Heatmap (D), GO (E), and KEGG pathway (F) analysis of differential miRNAs in CSF EVs from GBM patients and controls. Heatmap (G), GO (H), and KEGG pathway (I) of differential miRNAs expression between GBM and control tissue EVs.

The morphological characters of EVs isolated from GBM or control CSF were identified using TEM (Fig. 1A). Then Western blot analysis was used to test exosome marker CD63 (Fig. 1B). We examined the number and size distribution of GBM and controlled EVs using nanoparticle tracking analysis (NTA) (Fig. 1C). The result demonstrated that the mean diameter was 115.3 ± 42.6 nm for GBM CSF EVs and 119.6 ± 31.5 nm for control EVs.

### Differential expression of miRNAs and their characteristics in CSF EVs and tissues from GBM patients and controls

We analyzed miRNA profiles in CSF EVs from patients with pathologically confirmed GBM (n = 3) and non-tumor controls (n = 3) based on RNA-Seq. A total of 4,619 miRNAs were detected. We identified 26 significantly dysregulated miRNAs with a fold change ≥ 2 and P-value < 0.05 between GBM and control patient CSF EVs. 10 of the 26 miRNAs were significantly upregulated in GBM CSF EVs and 16 miRNAs were downregulated (Fig. 1D). Top 20 differentially expressed miRNAs in GBM and control CSF EVs are listed in Supplementary Table S1 (All differentially expressed miRNAs are listed in Supplementary Table S2). The target genes of the 26 differentially expressed miRNAs in CSF EVs were analyzed using Gene Oncology (GO) enrichment. The miRNA target genes mainly participated in binding (Fig. 1E, supplementary Figure S1 and S2). Kyoto Encyclopedia of Genes and Genomes (KEGG) pathway analysis showed that the differentially expressed miRNAs interacted with pathways, such as PI3K-Akt signaling NF-kappa B signaling (Fig. 1F). Even though we had identified the differential expressed miRNAs in CSF EVs, we still could not determine whether these miRNAs were specifically secreted from the GBM. Then, differentially expressed miRNAs between tumor tissues from GBM patients (n = 3) and brain tissues from cerebral trauma patients (n = 3) were detected based on RNA-Seq. There were 115 miRNAs considerably upregulated in GBM patients, and 130 miRNAs were significantly downregulated (Fig. 1G). Top 20 differential expressed miRNAs in GBM and control tissues are listed in Supplementary Table S3 (All differentially expressed miRNAs are listed in Supplementary Table S4). Then GO enrichment (Fig. 1H, supplementary Figure S1 and S2) and KEGG pathway (Fig. 1I) analysis were conducted to measure target genes of the 245 dysregulated miRNAs. We note that these differentially expressed miRNAs might be related to molecular binding in the cellular process and interact via several routes by GO and KEGG analysis.

### Identification of miRNA biomarker in CSF EVs of GBM patients

**Fig. 2:**
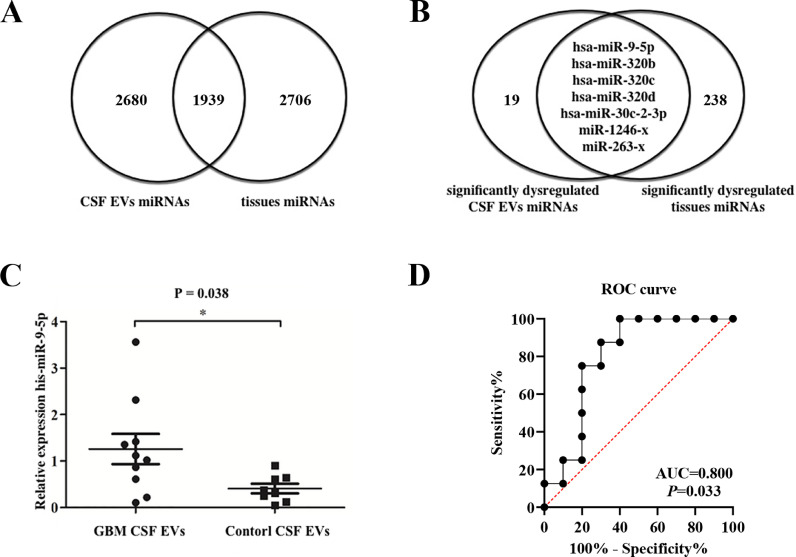
Identification miRNA biomarkers in CSF EVs of GBM patients. Venn diagram shows the total (A) and differentially expressed (B) miRNAs in CSF EVs overlap of tissues. (C) RT-qPCR shows that the expression level of miR-9 was significantly higher in GBM CSF EVs than controls (p = 0.038). (D) Receiver Operating Characteristics curve of miR-9.

There were 1,939 miRNAs detected in both CSF EVs and tissues (Fig. 2A). As mentioned above, 26 miRNAs were significantly dysregulated in GBM CSF EVs, while 245 miRNAs in GBM tissues. We identified seven significantly upregulated miRNAs in GBM CSF EVs, overlapping with tissue data (Fig. 2B), namely miR-9-5p, miR-320b, miR-320c, miR-320d, miR-30c-2-3p, miR-1246-x and miR-263-x. The results showed that the expression level of miR-9 was significantly higher in CSF EVs from GBM patients (n = 10) than those from control (n = 8) (Fig. 2C). The miR-9 expression level was also significantly higher in GBM tumor tissue. Receiver Operating Characteristics (ROC) curve was conducted to discriminate GBM and non-tumor control patients (Fig. 2D). The area under curve (AUC) for CSF EVs miR-9 was 0.800 (95%CI: 0.583-1.000, p = 0.033).

### Isolation and verification of GSCs and GSC-derived EVs

**Fig. 3:**
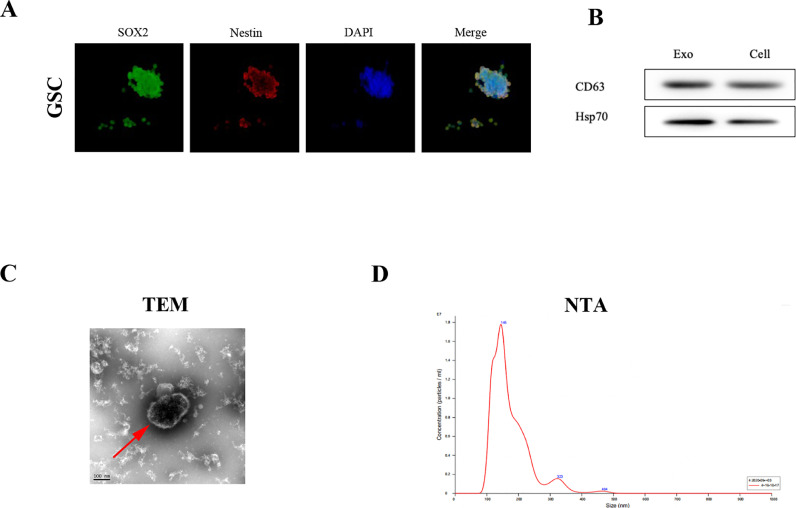
Isolation and verification of GSCs and GSC-derived EVs. (A) Immunofluorescence assay identified the GSCs surface markers, SOX2 (red) and Nestin (Green). (B) The purification of GSC-derived EVs was verified by the exosome markers CD63. (C) The morphology of GSC-derived EVs was visualized by transmission electron microscopy and (D) NTA analysis was taken to analyze the characterization of EVs.

Previous studies have demonstrated that glioma stem cells (GSCs) are a verified source of EVs, which cloud penetrate to GBM patients’ CSF through disturbed BBB. Therefore, we aimed at investigating the expression and role of miR-9 in GSC-derived EVs. The GSC1 and GSC2 primary cell lines were isolated from GBM patients’ surgery tissues and verified by tumorsphere formation assay (Fig. 3A). Two classical GSC markers, SOX2 and Nestin, were labeled in the tumorsphere by immunofluorescence (Fig. 3A), which indicated the successful purification of primary GSCs. Then, we collected and centrifuged the culture medium of GSC1 so as to get the EVs. And the EVs were verified by the surface marker CD63 (Fig. 3B), along with the shape in the TEM field (Fig. 3C) and the distribution of EVs by the NTA analysis (Fig. 3D).

### Targeting miR-9 in GSC-derived EVs for regulation of GBM cell growth

**Fig. 4:**
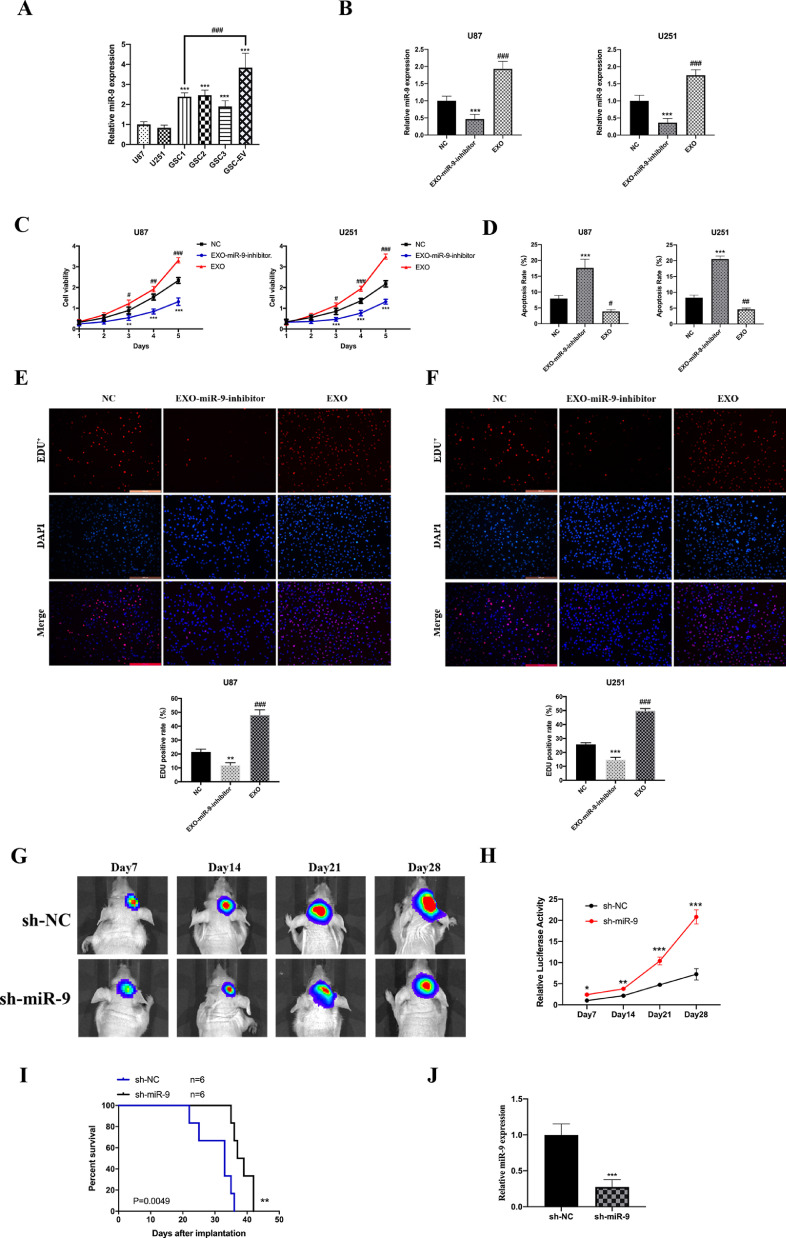
Targeting miR-9 in GSC-derived EVs for regulation of GBM cell growth. (A) The expression of miR-9 in GSC1/2 was significantly increased than U87 and U251 cell and showed strong enrichment in GSC-derived EVs than GSCs. (B) Exosomes isolated GSCs with or without miR-9 inhibitor treatment (termed as EXO and EXO-miR-9-inhibitor group) and then were co-incubated with U87 and U251 cells. The relative miR-9 expression was decreased after EXO-miR-9-inhibitor treatment and increased in EXO groups. (C) The cell growth was suppressed in EXO-miR-9-inhibitor groups while elevated in EXO groups both in U87 and U251cells. (D) Cell apoptosis was measured by flow cytometry. Cells treated with miR-9 inhibitor exosomes showed a higher apoptotic rate than control. The EdU positive cell rates was recorded. EXO groups showed increased tumor viability in U87 (E) and U251 (F) cells. (G and H) Intracranial xenograft model was employed. Inhibition of miR-9 impaired GBM growth and luciferase activity *in vivo*. (I) Kaplan-Meier analysis of overall survival of the sh-NC group and the sh-miR-9 group, n=6. (J)The expression of decreased in sh-miR-9 group. Data were represented as mean ± SD. *p < 0.05, **p < 0.01, ***p < 0.001 and ^#^p < 0.05, ^##^p < 0.01, ^###^p < 0.001 versus corresponding NC groups.

With the abundant collection of GSC-derived EVs, we first analyzed the expression of miR-9 in the EVs, GSCs and glioblastoma cell lines. The results showed that the miR-9 levels in GSC1, GSC2 and GSC3 were much higher than those in the U87 and U251 cells, while miR-9 in EVs from GSC1 was about 1.61-fold compared to miR-9 in GSC1 cell, which indicated an enriched miR-9 concentration (Fig. 4A). In order to investigate the role of GSC exosome miR-9 in GBM promotion, we treated GSC1 with miR-9 inhibitor for 48h and centrifuged the culture medium for the exosomes (termed as EXO-miR-9-inhibitor group). U87 and U251 cells were co-cultured with GSC-derived EVs (termed as EXO group), EXO-miR-9-inhibitor separately. Cells in the EXO groups showed a significantly increased miR-9 expression than control group, while the EXO-miR-9-inhibitor groups suppressed the miR-9 expression compared to the corresponding NC groups (Fig. 4B).

Next, the above three groups of U87 and U251 cells were underwent CCK8 and EdU experiments to detect cell growth in condition of GSC-derived EVs. The cell viability of EXO-miR-9-inhibitor groups was inhibited in both cell lines. In contrast, EXO groups showed an overt increase in cell growth as compared to controls (Fig. 4C). The U87 and U251 cells, which underwent GSC-derived EVs treatment, were then measured by flow cytometry for apoptosis analysis. The results showed that miR-9 inhibitor exosomes promoted cell death rate both in U87 and U251 cells (Fig. 4D). Meanwhile, the EdU assay revealed that EXO-miR-9-inhibitor groups exhibited decreased EdU positive cell rates and the EXO groups promoted in a statistically significant manner (Fig. 4E and 4F). In summary, the findings here suggested that miR-9 in GSC-derived EVs could be absorbed by glioblastoma cells and facilitated cell growth *in vitro*.

To further analyze the role of miR-9 in tumor growth *in vivo*, intracranial xenograft mice model was employed here. Briefly, the miR-9 short-hairpin RNA (sh-miR-9) and corresponding negative control shRNA (sh-NC) were transfected into GSCs, which were then implanted into the corpus striatum of mice brain. Intracranial tumor growth was measured by immunofluorescence every 7 days, along with the total survival time. Tumor volume with the fluorescence region was impeded in the sh-miR-9 group from day14, 21 and 28 (Fig. 4G and 4H). The survival percentage in the sh-miR-9 group was significantly prolonged compared to sh-NC group in Kaplan-Meier survival curves (Fig. 4I). The expression miR-9 in autopsy-derived tumors was measured and showed a significant level in sh-miR-9 group (Fig. 4J). Hence, we concluded that increased expression of miR-9 promoted GBM cell growth both *in vitro* and *in vivo*.

### MiR-9 in GSC-derived EVs augmented GBM cell migration

**Fig. 5:**
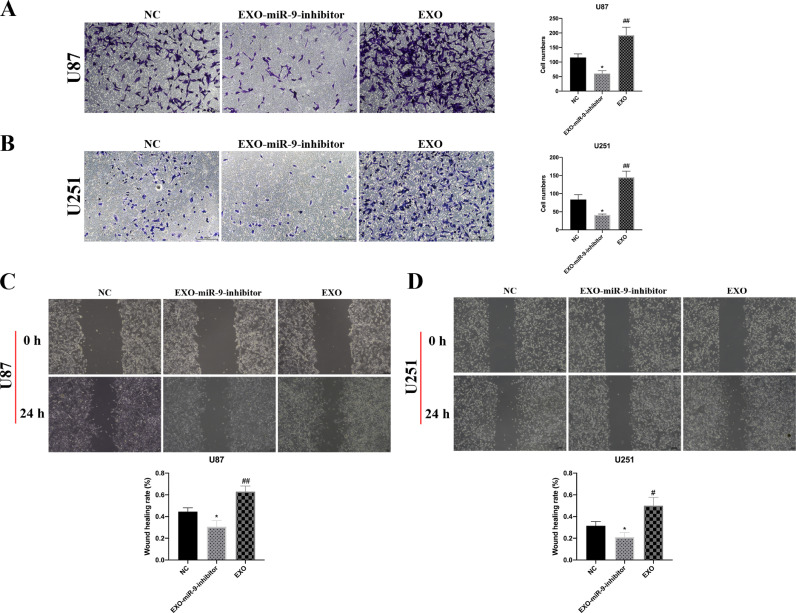
MiR-9 in GSC-derived EVs augmented GBM cell migration. (A) U87 cells treated with EXO-miR-9-inhibitor or EXO groups were underwent transwell analysis. The migrating cells were measured in five fields (200×) for an average number in one chamber. EXO administration increased penetrated cell numbers while miR-9 inhibitor showed the reverse effects. (B) The similar migration tendency in U251 cells was observed as in U87 cell. Wound healing assays of U87 (C) and U251(D) cells exhibited increased capacity of migration after treatment in EXO groups. All experiments were conducted at least 3 times. Data were represented as mean ± SD. *p < 0.05 and ^##^p < 0.01 versus corresponding NC groups.

U87 and U251 cells in the NC, EXO-miR-9-inhibitor and EXO groups were submitted to transwell and wound healing experiments. The migrated GBM cells were decreased in the EXO-miR-9-inhibitor group but incremental in the EXO group as compared to NC group (Fig. 5A and 5B). The ability of GBM cell wound healing was significantly restrained after treatment with miR-9 inhibitor related exosomes for 24 h (Fig. 5C and 5D). By contrast, the administration of GSC-derived exosomes could promote GBM cell penetration into the scratching wound in a signicifant manner after statistic quantification (Fig. 5C and 5D). These results demonstrated that miR-9 in the GSC-derived exosomes could increase GBM migration motility.

### MiR-9 promoted GBM malignant phenotypes potentially via regulation of DACT3

**Fig. 6:**
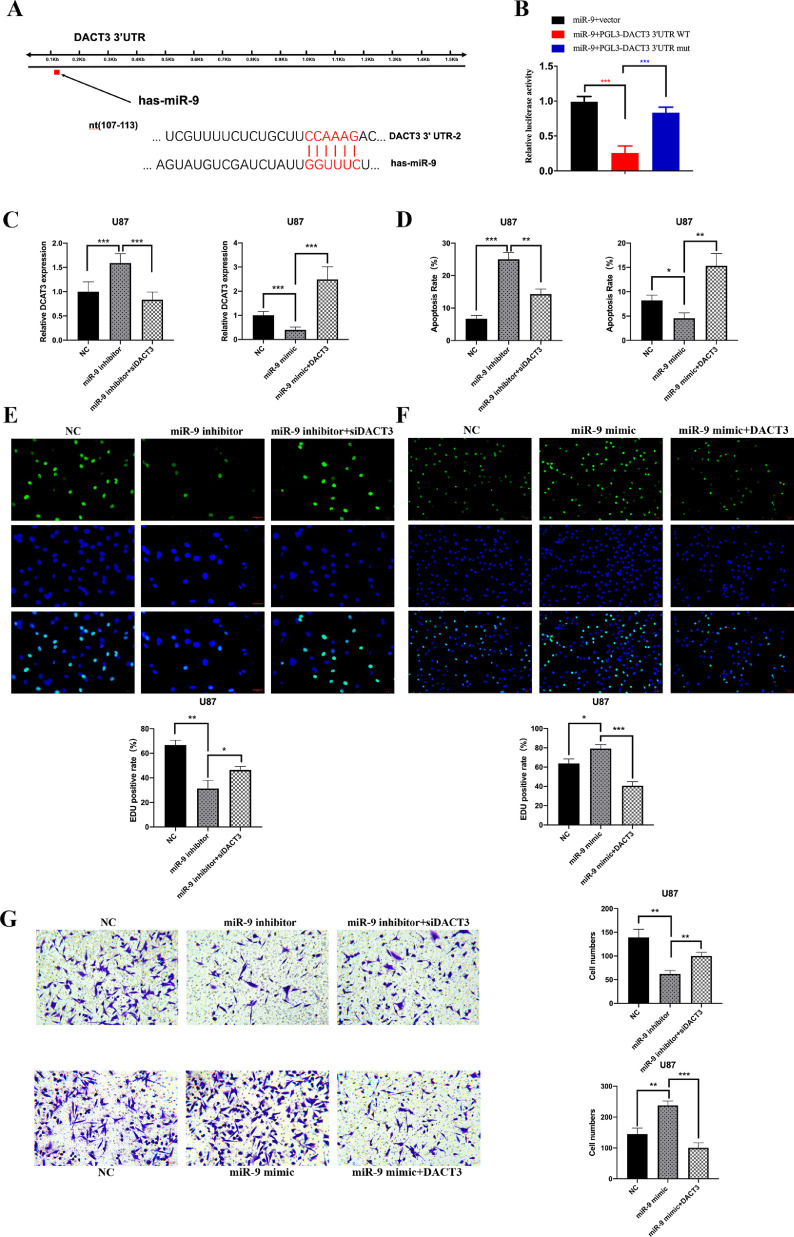
MiR-9 promoted GBM malignant phenotypes potentially via regulation of DACT3. (A)The predicted binding sequence between miR-9 and 3’UTR region of DACT3 mRNA. (B) MiR-9 decreased the luciferase activity in condition of wildtype binding sequence, while exerted no effects with mutant sequence. (C) In U87 cells, inhibition of miR-9 elevated the level of DACT3 and DACT3 siRNA suppressed its expression. (D) U87 cells cells in miR-9 inhibitor group showed a higher apoptosis rate, and the inhibitor+siDACT3 group suppressed cell apoptosis. This rate was reversed in the mimic and DACT3 vector group. (E and F) Treatment of miR-9 inhibitor restrained EdU positive cell rates, while additional administration of siDACT3 restored cell viability. Treatment of miR-9 mimic increased EdU positive cell rates, while additional administration of DACT3 vector impaired cell viability. (G) Treatment of miR-9 inhibitor impeded cell penetration in transwell assay and the migrated cells were increased in the miR-9 inhibitor+siDACT3 group. Treatment of miR-9 mimic promoted cell migration which was counteracted in the miR-9 inhibitor+siDACT3 group. The migrating cells were measured in five fields (200×) for an average number in one chamber. All experiments were conducted at least 3 times. Data were represented as mean ± SD. *p < 0.05, **p < 0.01, ***p < 0.001 versus corresponding control groups.

To identify potential targets of miR-9 which involved in GBM tumorigenesis, five bioinformatics websites (i.e., TargetScan, DIANAmT, miRanda, miRDB, and RNAhybrid) were analyzed. The collective results of the predictive websites suggested DACT3 as a promising downstream candidate gene of miR-9. DACT3 was demonstrated as a tumor suppressor in several malignant cancers, such as esophageal squamous cell carcinoma [22] and non-small cell lung cancer [23]. However, no research about DACT3 has been taken in gliomas. The miR-9 binding sequence of DACT3 gene 3’ UTR region was obtained from the bioinformatics websites and subsequent luciferase reporter assay was employed (Fig. 6A). The results showed that miR-9 administration pronouncedly inhibited luciferase activity in the DACT3 3’ UTR wildtype group and the inhibition was restored in condition of DACT3 3’ UTR mutation (Fig. 6B). These results suggested that miR-9 could directly bind with the 3’ UTR region of DACT3 and restrain its transcription.

In U87 cells, the mRNA level of DACT3 was increased after miR-9 inhibitor treatment while was suppressed with DACT3 siRNA administration. In contrast, miR-9 mimic treatment decreased DACT3 expression and the DACT3 vector restored its expression (Fig. 6C). The cell apoptosis was measured in the above groups. In brief, miR-9 inhibitor and DACT3 vector administration promoted cell apoptosis. The anti-apoptosis effect of miR-9 mimic could be restored by additional DACT3 treatment (Fig. 6D). MiR-9 inhibitor could decrease U87 cell growth compared to control group, while inhibition of DACT3 could elevate the EdU positive cell rate after miR-9 inhibitor treatment in EdU proliferation experiment. The miR-9 overexpression by mimic treatment promoted U87 cell viability while the overexpression of DACT3 could counteract the miR-9 upregulation (Fig. 6E and 6F). The transwell assays showed the same tendency as EdU positive rates, in which DACT3 siRNA restored the impaired migration ability induced by low expression of miR-9. The administration of miR-9 mimic and DACT3 vector showed the reverse results than in the above experiment (Fig. 6G). Therefore, we could draw a conclusion that miR-9 overexpression promoted GBM cell proliferation and migration through DACT3 inhibition and the miR-9/DACT3 axis might be a potential mechanism in GBM malignant phenotypes.

## Discussion

The presence of specific cargo of EVs in the CSF can carry cancer molecular and genetic mutations, which have the potential to become the accessible source of biomarkers, contributing to diagnosis and prognostication of brain tumors. Using RNA-seq and bioinformatics methods, we have identified 26 differentially expressed EV miRNAs in CSF that discriminate GBM patients from non-tumor controls. Liquid biopsy is a hot research area in recent years, GBM-derived EVs had been detected in patients’ serum. Saeideh et al. [24] reported 21 significant differentially expressed miRNAs in serum exosomes from GBM patients relative to the healthy population, however, the miRNA expression profiles differred between CSF and serum EVs. A previous study also revealed substantial differences in exosomal miRNA profiles between healthy human CSF and serum [25]. CSF is intrinsically regulated at a higher homeostasis level [26], while EV contents of blood are easily influenced by clinical variables such as dietary intake or blood pressure [27]. This may make CSF EVs more attractive as a biomarker development platform.

In GBM tissues, 245 differentially expressed miRNAs were identified compared with non-tumor tissues. 6 miRNAs significantly upregulated in both GBM CSF and tissue EVs were detected. Yagi et al. [25] also reported that not all miRNAs released from the normal brain could be detected in CSF EVs and that the miRNA expression profiles of normal brain tissue and CSF EVs were not identical. This suggests that EVs from GBM cells can selectively package miRNA species and release to CSF via a distinct pathway [28]. Shi et al. [29] reported that miR-21 in CSF EVs of GBM patients was upregulated compared to controls. The differentially expressed miRNA, miR-9, identified in our CSF EVs, has not been previously identified in other studies. It is likely due to differences in miRNA detection and data analysis methods [30]. In addition, the EV-containing miR-9 in serum and CSF of acute ischemic stroke patients, which gave us a hint in searching GBM CSF biomarker.

In the present study, the expression level of CSF miR-9 predicted a preoperative GBM diagnosis with 80.0% accuracy. Chen et al. [31] reported that miR-9 is frequently upregulated in human glioma tissues and cells and functions as an onco-miRNA. MiR-9 is secreted from glioblastoma exosomes and then absorbed by vascular endothelial cells, leading to increased angiogenesis. Therefore, our results showed that the levels of miR-9 in CSF EVs could be demonstrated as a promising indicator for GBM diagnosis.

It remains challenging to obtain sufficient amounts of CSF EVs from patients with giant GBM due to the safety of performing a lumbar puncture before the surgery. Previous studies reported that performing lumbar punctures in patients with brain tumors was generally safe [8,32]. In the future, abundantly available EVs might be isolated from tiny amounts of CSF due to the adoption of more high and straightforward efficiency approaches [33]. It might help make CSF collection much safer.

The secretion of molecules and genetic substance via GSC-derived EVs plays a vital role in GBM progression, which might exert transcriptional and epigenetic modifications in tumor initiation and resistance to therapies [15]. Here, we found increased expression of miR-9 in GSCs compared with GBM cell lines. Wu et al. [34] reported whole RNA-seq data of four GSC-EVs, of which miR-9 expression exhibited strong enrichment. This may lead to a hypothesis that GSCs could secret miR-9 to the peripheral glioma cells and the intermediate would be the exosomes. It was proved here the much higher level of miR-9 in the purified GSC-EVs. As previously reported, GSC-derived EVs functioned as a significant way for the intercellular communication [19]. Astounding numbers of coordinated and plastic interactions between GSCs and surrounding environment exerted resistance to therapeutic interventions via exchange of EVs. In addition, novel biomarkers contained in GSC-EVs could be detected in circulating liquid biopsy, such as serum and CSF, which reflected the gene mutations, heterogeneity states and therapeutic responses [35]. Moreover, if EVs could be released from GSCs and penetrate BBB, exogenous synthesis of exosomes injected to CSF would take effects in GBM through the fluxion of CSF. This would be a promising strategy to deliver therapeutic inhibitors directly to CNS tumor microenvironment. Recently, mesenchymal stem cell (MSC)-derived EVs have been considered as a possible treatment for many CNS diseases, which can be absorbed by tumor cells and participate in its malignant behavior. This has implications for targeting CSF-EVs as a safer and more effective treatment method of GBM.

DACT3 is a bona fide tumor suppressor in many malignancies. In NSCLC cells, the expression of DACT3 reduced c-Myb expression, thus decreasing the activation of Wnt/β-catenin signaling pathway and inhibiting proliferative and invasive capacity [23]. As well as in breast cancer cells, depletion of DACT3 promote cell autophagy and tumorigenesis [22]. However, the role of DACT3 in suppression of GBM was not well illustrated as previously reported. We here found that miR-9 bound with the 3’UTR region of DACT3 mRNA and decreased its expression. Inhibition of DACT3 by relative siRNAs significantly facilitated GBM cell growth and migration. However, the elaborate pathway involving DACT3 in GBM needed further research.

## Conclusions

In the present study, we showed that miR-9 could serve as a novel biomarker in the CSF EVs for the diagnosis of GBM, while GSCs had been confirmed as reliable source of secreted exosomes. The expression of miR-9 was increased in GSCs and GSC-derived EVs compared to the differentiated cells, suggesting the EV-mediated miR-9 transfer from GSCs to GBM cells. Inhibition of miR-9 in GSC-EVs suppressed the GBM malignant phenotypes via the regulation of DACT3. These findings indicate that functional EVs containing miR-9 will be a promising candidate for diagnosis and treatment of glioblastoma in future clinical settings.

## Authors' contributions

All authors contributed to the study conception. This original work was mainly designed by Ning Liu and Jing Ji. Material preparation, data collection and analysis were performed by Liangyuan Geng, Jinjin Xu, Yihao Zhu, Xinhua Hu, Yong Liu, Kun Yang, Hong Xiao, Yuanjie Zou and Hongyi Liu. The first draft of the manuscript was written by Liangyuan Geng and Jinjin Xu. Statistical analysis was done by Yihao Zhu. All authors commented on previous versions of the manuscript. All authors read and approved the final manuscript.

## Ethics approval and consent to participate

Ethics Committee approved this project (Ethical Approval No. 2018-KY014-01) of the Affiliated Nanjing Brain Hospital, Nanjing Medical University (Chairperson, Professor Hongyi Liu) on February 28, 2018. All of the methods were carried out following the approved guidelines and the Declaration of Helsinki.

## Consent for publication

Written informed consent was obtained from all of the patients before surgery for publication of the research.

## Funding

The present study was supported by the General Project of Nanjing Medical Science and Technology Development (Grant No. YKK18185) and the National Natural Science Foundation of China (Grant No. 81972153).

## Availability of data and materials

The datasets used and/or analyzed during this study are available from the corresponding author on reasonable request.

## Declaration of competing interest

We declare that we have no financial and personal relationships with other people or organizations that can inappropriately influence our work, there is no professional or other personal interest of any nature or kind in any product, service and/or company that could be construed as influencing the position presented in, or the review of the manuscript.

## Appendix Supplementary materials

Image, application 1

Image, application 2

Image, application 3

Image, application 4

Image, application 5

Image, application 6
