# Supplementary material for: Targeting miR-9 in Glioma Stem Cell-Derived Extracellular Vesicles: A Novel Diagnostic and Therapeutic Biomarker
Source: Transl Oncol. 2022 May 19;22:101451. doi: 10.1016/j.tranon.2022.101451 (PMC9126959; doi:10.1016/j.tranon.2022.101451)
Supplement: Supplementary file 1 [file mmc1.docx]

| **Table S1. Top 20 differential expressed miRNAs in GBM and control CSF EVs** | | | | |
| --- | --- | --- | --- | --- |
| **miRNAs** | **GBM mean** | **Control mean** | **log2(fc)** | **P Value** |
| miR-1895-y | 158.510 | 0.010 | 13.952 | 0.020 |
| hsa-miR-144-5p | 121.120 | 0.010 | 13.564 | 0.048 |
| novel-m0972-3p | 100.021 | 0.010 | 13.288 | 0.025 |
| miR-1246-x | 90.486 | 0.010 | 13.143 | 0.025 |
| novel-m0304-3p | 79.267 | 0.010 | 12.953 | 0.013 |
| miR-4516-x | 68.864 | 0.010 | 12.750 | 0.018 |
| hsa-miR-432-5p | 60.541 | 0.010 | 12.564 | 0.036 |
| hsa-miR-30c-2-3p | 54.128 | 0.010 | 12.402 | 0.025 |
| hsa-miR-9-3p | 1550.738 | 7.896 | 7.618 | 0.004 |
| hsa-miR-320d | 2534.078 | 43.319 | 5.870 | 0.037 |
| miR-263-x | 158.445 | 4185.812 | -4.723 | 0.019 |
| miR-8109-x | 181.449 | 6734.143 | -5.214 | 0.029 |
| miR-12321-x | 3.771 | 246.828 | -6.033 | 0.049 |
| miR-252-x | 4.711 | 506.159 | -6.747 | 0.042 |
| miR-11240-x | 1.881 | 334.251 | -7.473 | 0.035 |
| miR-2547-y | 0.010 | 52.818 | -12.367 | 0.045 |
| miR-8859-y | 0.010 | 123.987 | -13.598 | 0.047 |
| miR-4638-y | 0.010 | 534.340 | -15.705 | 0.021 |
| miR-10316-x | 0.010 | 815.843 | -16.316 | 0.038 |
| miR-8490-y | 0.010 | 844.723 | -16.366 | 0.037 |
