# Supplementary material for: Targeting miR-9 in Glioma Stem Cell-Derived Extracellular Vesicles: A Novel Diagnostic and Therapeutic Biomarker
Source: Transl Oncol. 2022 May 19;22:101451. doi: 10.1016/j.tranon.2022.101451 (PMC9126959; doi:10.1016/j.tranon.2022.101451)
Supplement: Supplementary file 2 [file mmc2.docx]

| **miRNAs** | **GBM mean** | **Control mean** | **log2(fc)** | **P Value** |
| --- | --- | --- | --- | --- |
| novel-m1358-5p | 9.112 | 0.010 | 9.832 | 0.000 |
| hsa-miR-216a-3p | 5.071 | 0.010 | 8.986 | 0.000 |
| hsa-miR-4421 | 4.907 | 0.010 | 8.939 | 0.000 |
| hsa-miR-216b-3p | 4.800 | 0.010 | 8.907 | 0.004 |
| miR-615-y | 4.331 | 0.010 | 8.759 | 0.001 |
| hsa-miR-4707-3p | 1.788 | 0.010 | 7.482 | 0.034 |
| miR-532-y | 1.784 | 0.010 | 7.479 | 0.001 |
| hsa-miR-10b-3p | 40.514 | 0.254 | 7.319 | 0.000 |
| miR-3074-x | 1.479 | 0.010 | 7.208 | 0.006 |
| hsa-miR-10a-3p | 1.423 | 0.010 | 7.153 | 0.010 |
| miR-144-x | 0.010 | 1.131 | -6.821 | 0.016 |
| novel-m0762-3p | 0.010 | 1.272 | -6.991 | 0.010 |
| novel-m0836-3p | 0.010 | 1.272 | -6.991 | 0.010 |
| miR-656-y | 0.010 | 1.274 | -6.993 | 0.028 |
| miR-33-x | 0.010 | 1.409 | -7.139 | 0.006 |
| hsa-miR-1258 | 0.010 | 1.487 | -7.216 | 0.004 |
| novel-m0722-3p | 0.010 | 1.634 | -7.352 | 0.014 |
| miR-935-y | 0.010 | 2.662 | -8.056 | 0.001 |
| hsa-miR-499a-3p | 0.010 | 3.858 | -8.592 | 0.000 |
| hsa-miR-642b-3p | 0.010 | 10.623 | -10.053 | 0.003 |
|  | | | | |

| **Table S3. Top 20 differential expressed miRNAs in GBM and control tissues** |
| --- |
